# Exploring the Potential of a Novel Iodine-Based Material as an Alternative Contrast Agent in X-ray Imaging Studies

**DOI:** 10.3390/ma17092059

**Published:** 2024-04-27

**Authors:** Kristina Bliznakova, Iliyan Kolev, Nikolay Dukov, Tanya Dimova, Zhivko Bliznakov

**Affiliations:** 1 Department of Medical Equipment Electronic and Information Technologies in Healthcare, Faculty of Public Health, Medical University of Varna, 9002 Varna, Bulgaria; ntdukov@mu-varna.bg (N.D.); zhivko.bliznakov@mu-varna.bg (Z.B.); 2 Department of Pharmaceutical Chemistry, Faculty of Pharmacy, Medical University of Varna, 9002 Varna, Bulgaria; ilian.kolev@mu-varna.bg (I.K.); tania.dimova@mu-varna.bg (T.D.)

**Keywords:** radiocontrast agent, X-ray imaging, physical phantoms, simulations

## Abstract

Background: Contrast-enhanced mammography is one of the new emerging imaging techniques used for detecting breast tissue lesions. Optimization of imaging protocols and reconstruction techniques for this modality, however, requires the involvement of physical phantoms. Their development is related to the use of radiocontrast agents. This study assesses the X-ray properties of a novel contrast material in clinical settings. This material is intended for experimental use with physical phantoms, offering an alternative to commonly available radiocontrast agents. Materials and Methods: The water-soluble sodium salt of the newly synthesized diiodine-substituted natural eudesmic acid, Sodium 2,6-DiIodo-3,4,5-TriMethoxyBenzoate [NaDITMB], has been investigated with respect to one of the most commonly applied radiocontrast medium in medical practice—Omnipaque^®^. For this purpose, simulation and experimental studies were carried out with a computational phantom and a physical counterpart, respectively. Synthetic and experimental X-ray images were subsequently produced under varying beam kilovoltage peaks (kVps), and the proposed contrast material was evaluated. Results and Discussion: Simulation results revealed equivalent absorptions between the two simulated radiocontrast agents. Experimental findings supported these simulations, showing a maximum deviation of 3.7% between the image gray values of contrast materials for NaDITMB and Omnipaque solutions for a 46 kVp X-ray beam. Higher kVp X-ray beams show even smaller deviations in the mean grey values of the imaged contrast agents, with the NaDITMB solution demonstrating less than a 2% deviation compared to Omnipaque. Conclusion: The proposed contrast agent is a suitable candidate for use in experimental work related to contrast-enhanced imaging by utilizing phantoms. It boasts the advantages of easy synthesis and is recognized for its safety, ensuring a secure environment for both the experimenter and the environment.

## 1. Introduction

Contrast-enhanced imaging of the breast, implemented in mammography, tomosynthesis, computed tomography (CT), and magnetic resonance imaging (MRI), is an imaging technique used for detecting breast tissue abnormalities via the combination with iodine-based contrast agents. The latter is used to improve diagnostic accuracy through the detection of areas of increased vascularization in the breast [1,2]. It uses contrast in order to enhance the visualization of tumor neovascularity [3]. Studies on contrast-enhanced mammography (CEM), also called contrast-enhanced spectral mammography (CESM) or contrast-enhanced digital mammography, demonstrated that the sensitivity of this new modality ranges from 93% to 100%, while specificity is within the range of 63% to 88% [4,5,6,7,8].

The used contrast material is a key element of CESM and essential for the optimization of the imaging technique as well as the imaging protocol of this modality. For instance, an optimization task focuses on studying detectors that have optimal characteristic X-ray emissions within the suitable energy range used for breast imaging [9]. Another optimization objective is the evaluation of the minimum contrast medium needed for CESM [10] as well as quantifying the radiation dose in CESM as it varies with breast density and among specific patient subpopulations appropriate for this examination [11]. Up-to-date, there are no definitive studies yet published documenting both the optimal imaging parameters for CEM/CESM and the optimal concentration of contrast material used, although generally accepted guidelines exist [3].

When optimizing and evaluating the technological parameters of contrast-enhanced imaging systems, physical phantoms play a critical role [12]. These specialized phantoms, designed for research in contrast-enhanced imaging, utilize materials to correctly replicate the radiological characteristics of both soft tissues and radiocontrast agents. While studies on the representation of breast tissue using different materials are actively pursued, the same is not valid for the radiocontrast agents. The majority of phantom implementations include the use of contrast agents commonly employed in clinical practice [10,13,14,15]. Other researchers, such as Carton et al. [16], used a homogeneous polymethyl methacrylate phantom containing iodine disks with an iodine areal concentration of 0.5, 1, 2, 4, 10, and 20 mg /cm^2^ in order to assess the conspicuity of the iodine in novel three-dimensional (3D) imaging technique.

Overall, there is a growing interest in ongoing research aiming at developing novel contrast agents, specifically for CT applications, which may lead to enhancing imaging quality, minimizing side effects, targeting specific tissues, and enabling precise imaging of diseased areas. These advancements, in contrast agents play a crucial role in disease detection and monitoring by enabling early-stage detection and facilitating timely intervention and treatment. For instance, graphene oxide has emerged as a potential contrast agent for micro-CT, offering the capability to visualize fat within and around the heart [17]. Recent studies have also introduced innovative contrast agents such as iopamidol loaded in chitosan nanospheres modified by Anti-5-HT3R antibody for intestinal-targeted imaging [18]. Researchers have also demonstrated significant interest in metallic bismuth nanoparticles for medical applications [19]. Rizzo et al. [20] explored a novel bismuth-based CT contrast agent based on the HPDO3A ligand, leveraging bismuth’s exceptional X-ray absorption properties, making it an ideal candidate for contrast agent design. Furthermore, Amato et al. [21] highlighted the need for novel contrast agents, particularly those based on bismuth, given the limitations of iodine and barium in certain imaging scenarios due to their relatively low energy absorption properties. Their research revealed a promising bismuth-based contrast agent suitable for preclinical applications, demonstrating superior contrast compared to iodine agents [22]. In the field of breast imaging, a review paper underlines the significance of nanoparticles, including gold, platinum, and silver, as highly beneficial contrast agents. Their adaptability and flexibility to modification make them invaluable for contrast-enhanced breast imaging. As highlighted by Waller et al. [23], while mammography techniques leveraging enhanced tissue contrast show promise, the development of contrast agents tailored for this methodology is still in its nascent stages. All preclinical studies were performed on phantoms and mice. At the same time, the utilization of high-Z elements for contrast-enhanced CT applications beyond iodine presents challenges in terms of biocompatibility. While toxicological data have been gathered, its adequacy for immediate clinical use remains uncertain.

When using iodine-based contrast in optimization studies, it becomes essential for its radiological properties to closely resemble those of the clinically employed contrasts. In most medical departments, these radiocontrast agents are primarily dedicated to clinical examinations, making their application in the optimization of imaging protocols challenging and often unfeasible. Furthermore, the cost associated with these clinical radiocontrast agents is high, especially when a series of experiments need to be conducted [24]. This imposes the exploration of new alternative iodine-based contrast materials that exhibit X-ray characteristics close to those of the clinical counterparts while also being easily synthesizable within a home laboratory, which is, in fact, the aim of this study.

This work investigates the feasibility of a newly developed iodine-based radiocontrast material for research applications in X-ray imaging. The long-term goal of the research work of the ELPIDA group at the Medical University of Varna is focused on the development of a prototype system for contrast-enhanced breast tomography. The novel contrast is specifically intended for use with physical phantoms, which will be exploited in extensive experimental work related to optimizing acquisition geometry and related reconstruction algorithms specifically tailored for contrast-enhanced imaging modalities. The phantoms with the contrast will also be used to compare newly developed modalities to currently available mammography imaging systems.

## 2. Materials and Methods

The study approach included the use of a computational simulation study, followed by an experimental work. Previously, our team reported on a newly synthesized 2,6-diiodo-3,4,5-trimethoxybenzoic acid, also known by the acronym DITMBA [25,26]. The elemental composition of DITMBA was an input for the computational study aimed at initially assessing its X-ray properties with respect to the iodine-based contrast materials commonly utilized in clinical and experimental settings. In order to confirm and validate the findings of the theoretical experiment, the water-soluble sodium salt of the acid in question, NaDITMB, was also prepared; а form that actually mimics the pharmaceutical forms of commercial radiopaque agents used.

### 2.1. Computational Study

**Figure 1:**
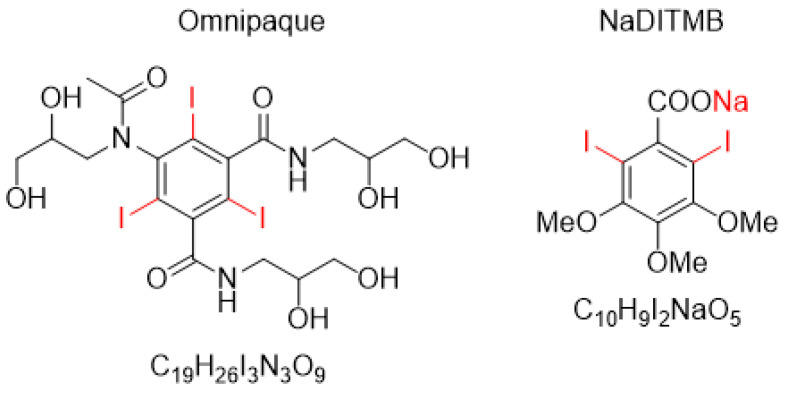
Radiocontrast agents used in the computational study.

The molecular structures of the investigated contrast agents are shown in Figure 1. They include Omnipaque, GE Healthcare, Shanghai, China, a radiocontrast agent currently used in clinical practice, as well as the newly obtained NaDITMB, Medical University of Varna, Varna, Bulgaria [25,26].

**Figure 2:**
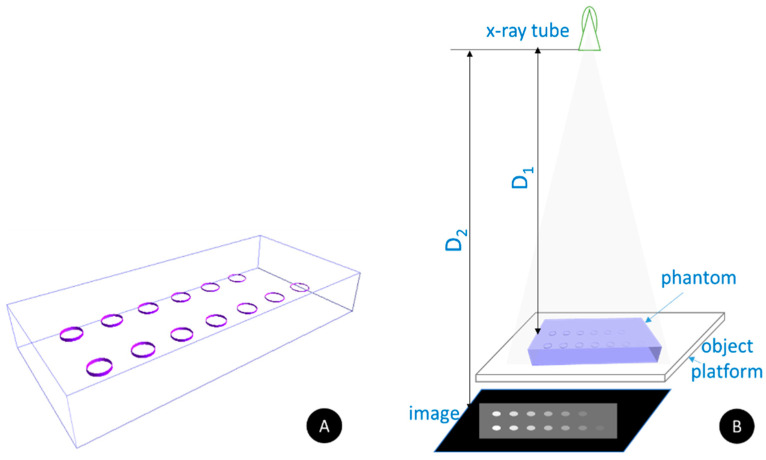
Computational study: (**A**) A Plexiglas box with a thickness of 45 mm from resin with 14 cylindrical objects with a radius of 10 mm and thickness, which varies between 0.2 mm and 2.6 mm. Each cylinder is filled with a contrast agent of the same type. Two such phantoms were created, with the following two contrast agents, contained in (i) iohexol (Omnipaque) and (ii) sodium 2,6-diiodo-3,4,5-trimethoxybenzoate (NaDITMB); (**B**) Geometry used to generate synthetic X-ray projection images. D_1_ and D_2_ are the distances between the X-ray tube and the surface of the object placed on a platform and the X-ray tube and the detector, respectively. These distances are 100 cm and 130 cm, respectively.

A computational phantom was designed, comprising of 14 cylindrical objects with varying heights placed within a monolithic Plexiglas block, as shown in Figure 2A. The phantom has a thickness of 45 mm and, together with its composition, mimics well the X-ray absorption properties of the breast tissue with a composition of 50% adipose and 50% glandular tissues. The selected thickness corresponds to the average composition of a breast, compressed to 45 mm [27,28]. The 14 cylindrical objects have a radius of 10 mm, while the cylinder thickness ranges from 0.2 mm to 2.6 mm. Every individual cylinder was filled with a radiocontrast agent. The creation of the phantom and the generation of its X-ray projection images are facilitated by the utilization of the in-house developed XRAYImagingSimulator [29], which is an application (developed by Kristina Bliznakova as part of her Ph.D. Thesis) designed for modeling diverse phantoms in terms of shape and realism, encompassing anthropomorphic variations. It facilitates their integration into virtual imaging trials involving various X-ray imaging modalities. The software has several modules: (a) a module for the creation of computational phantoms either by simple uniform geometrical shapes (i.e., parallelepipeds, cylinders, ellipsoids, cones, etc.) with user-defined dimensions, orientation, and composition, or based on patient DICOM images from 3D imaging modalities, such as CT, MRI, which results in voxel-based phantoms; (b) a visualization module: for X-ray images, tomographic slices and the created 3D computational phantom; (c) modules for simulation of planar and tomographic X-ray imaging, both implemented either analytically [29] or via Monte Carlo [30]. For the simulations, each solid object and every voxel is characterized by elemental composition, which is needed for the calculation of the X-ray attenuation coefficients. For instance, an anthropomorphic breast phantom with a realistic external shape and internal structures may be represented by a semi-ellipsoid for its external contour, coupled with a series of cylinders for the glandular tissue, and a combination of intersecting ellipsoids for the adipose and Cooper tissues [31]. In this study, we employed the analytical approach in generating X-ray images, which is based on the Lambert-Beer law (Equation (1)), to model the attenuation of X-rays as they traverse the computational phantom with specified elemental compositions (Figure 2B). Further, the X-ray tracing method is employed to calculate the trajectory of each X-ray as it traverses through different structures.

For comparative analysis, we used Omnipaque, which is clinically utilized with CEM. This substance was used to initially assess the performance of our contract. In both models, the iodine concentration is held constant at 23.35 mg/mL, yielding the following solutions: (a) 5.03% iohexol (by weight) + 94.97% H_2_O (by weight), and (b) 4.47% NaDITMB (by weight) + 95.53% H_2_O (by weight). The mass attenuation coefficients sourced from the NIST XCOM database [32] are integrated into the software application.

Two different computational phantoms were created, differing in the elemental composition of the cylindrical objects, which were iohexol 23.35 mg I/mL and NaDITMB. Subsequently, the X-ray absorption characteristics of these radiocontrast agents were mathematically modeled and used with XRAYImagingSimulator to generate X-ray projection images by implementing the geometry shown in Figure 2B. For the simulation, we used two X-ray incident beam kilovoltage peaks (kVps), one corresponding to mammography irradiation at 35 kVp, derived from an X-ray tube with a Rh/Rh target/filtration combination (with an average energy of 20 keV) [33] and another X-ray spectrum obtained at 100 kVp from a W/Al target/filtration combination (with an average energy of 51 keV) [34,35]. Simulated distances were the following: the distance between the X-ray tube and the surface of the object (D_1_) is 100 cm, and the distance between the X-ray tube and the detector (D_2_) is 130 cm. Images were with dimensions of 4000 × 4000 pixels, each pixel with a size of 0.1 mm (square form). The values of the pixels in the X-ray image represent a line-integral—*μ*(*x*,*y*,*z*).*dl*, part of the Beer’s law:

(1) $$I=I_{0}*exp\left(-\int_{l}\mu (x,y,z)\right)dl$$

In the above equation, *µ*(*x*,*y*,*z*) represents the contrast solution’s linear attenuation coefficient that varies spatially at a given energy, while *l* indicates the path length traversing the object. Additionally, *I*_0_ denotes the radiation intensity originating from the source segment, uniformly emitted across the detector’s area for all viewing angles, while I is the radiation intensity reaching the detector. In this study, the X-images are shown as line integrals. More specifically, the *µ* of the contrast solution is represented as:

(2) $${\mu (E)}_{solution}=\left(w_{x}{\left(\frac{\mu \left(E\right)}{\rho }\right)}_{x}+w_{water}{\left(\frac{\mu \left(E\right)}{\rho }\right)}_{water}\right)\rho_{solution}$$

where the index *x* refers either to iohexol or NaDITMB, denoting the substance in consideration. The symbol *w* represents the weighting coefficient of the corresponding substance, indicating its contribution to the solution. Further, *ρ_solution_* is the measured density of the contrast solution, while $\left(\frac{\mu \left(E\right)}{\rho }\right)$ is the mass attenuation coefficient of the corresponding substance for a given X-ray energy, *E*. 

### 2.2. Experimental Study

(1) Synthesis of 2,6-diiodo-3,4,5-trimethoxybenzoic acid [DITMBA] and its sodium 2,6-diiodo-3,4,5-trimethoxybenzoate solution [NaDITMB]

**Figure 3:**
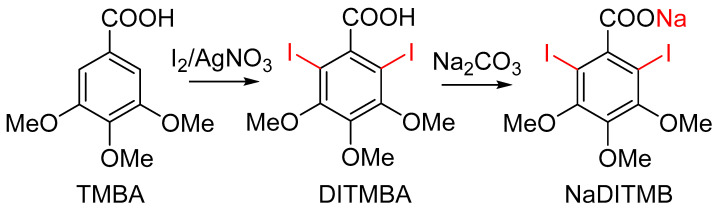
Steps in the synthesis of NaDITMB.

**Figure A1:**
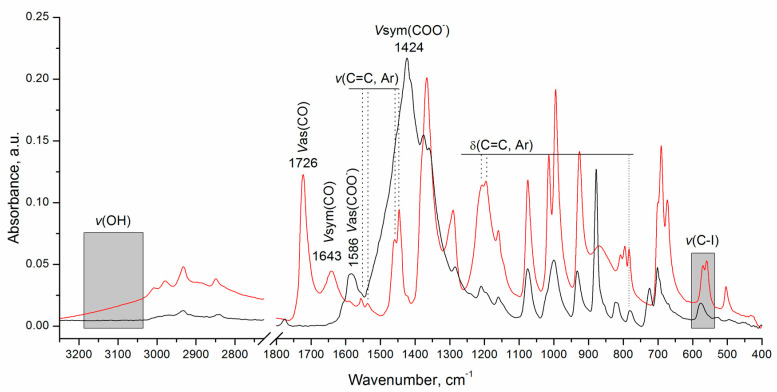
ATR FTIR analysis of NaDITMB (black color) and DITMB (red color).

The synthesis of DITMBA was performed according to the procedure described in [25]. In brief, stoichiometric amounts of each reagent were placed in a one-necked round-bottom flask and dissolved in anhydrous methanol. The reaction mixture was stirred for 24 hours at room temperature. The desired product, DITMBA, was separated from the inorganic residue and precipitated in water. The acid in question recrystallizes from boiling water; yield >95%, purity >98%. For the purposes of the intended comparative X-ray analysis, the resulting acid was converted into its water-soluble sodium salt, NaDITMB (Figure 3). In addition, attenuated total reflectance Fourier Transform Infrared (ATR FTIR) spectra of NaDITMB and DITMBA were recorded on a Bruker Tensor II FTIR spectrometer, Billerica, MA, USA in the 4000 ÷ 400 cm^−1^ spectral range. Both spectra are presented in Figure A1 in the Appendix A part.

The NaDITMB solution was prepared by applying the classical method of dissolving water-insoluble carboxylic acids in an aqueous-alkaline medium. To this aim, 64 mg of DITMBA and 7.3 mg Na_2_CO_3_ were dissolved in 0.100 mL of water under vigorous magnetic stirring (at ~250 rpm). The so-obtained aqueous solution has been then transferred into a small lightproof vial. The sample was stored at 4 °C in the dark (i.e., in the absence of solar or artificial ultraviolet-visible radiation).

(2) X-ray imaging

**Figure 4:**
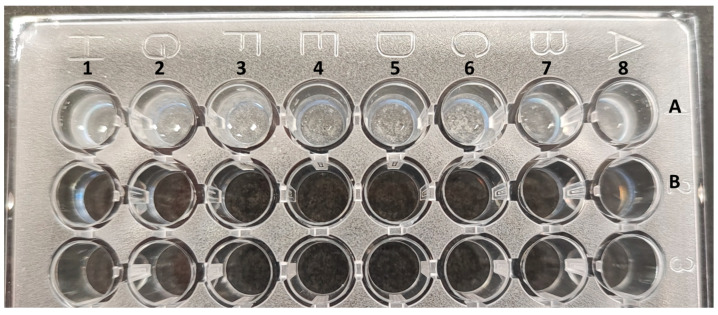
The plastic container with the iodine-based agents: A—NaDITMB, B—Omnipaque. The volumes of solutions in cylindrical cavities 1 to 3 were 0.2 mL, while those in cavities 4 to 8 were 0.1 mL each, maintaining a constant iodine concentration.

A standard plastic 96-well cell culture plate (Corning^®^, Corning, NY, USA flat bottom) was utilized as a template to house the working contrast solutions, as shown in Figure 4. The arrangement of the radiocontrast substances is in two consecutive rows within the plate. Row 1 (identified as ‘A’ in Figure 4) contained NaDITMB at a concentration of 50.3 mg I/mL, while row 2 (identified as ‘B’) accommodated Omnipaque at 44.685 mg I/mL. Each cylindrical cavity uniformly distributed iodine at a consistent concentration of 23.35 mg /mL. The volumes of solutions in cylindrical cavities 1 to 3 were 0.2 mL, while those in cavities 4 to 8 were 0.1 mL each, maintaining a constant iodine concentration. The densities of the solutions, determined to be 1.04 g/cm³, were measured using the Handheld Density Meter, Mettler Toledo, Columbus, OH, USA.

The plastic container, loaded with the contrast agents, was scanned with a radiographic system (Philips Juno DRF, Philips, Amsterdam, The Netherlands) under various imaging conditions: 46 kVp, 80 kVp, 95 kVp, and 100 kVp, by using the automatic exposure control (AEC) option. The planar X-ray images are the size of 1240 pixels × 380 pixels, with a pixel size of 0.148 mm × 0.148 mm. These kVps are used in daily radiology practice to obtain radiology images of human tissues. The source-to-object distance was 100 cm, while the source-to-detector distance was 110 cm.

## 3. Results and Discussion

### 3.1. Simulation Results

**Figure 5:**
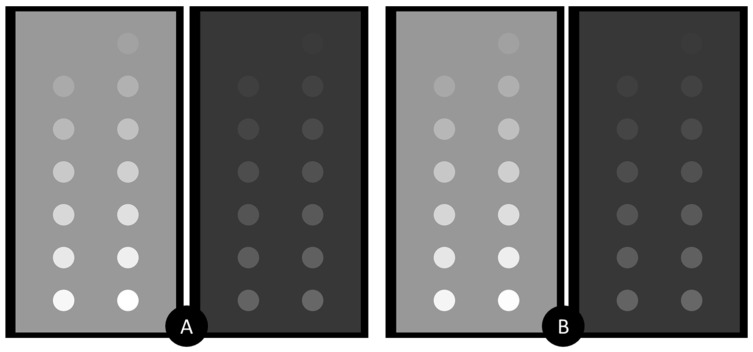
Analytical comparison of the X-ray attenuation properties of the two studied iodine-based materials: (**A**) X-ray image of the phantom with NaDITMB, (**B**) X-ray image of the phantom with Omnipaque. The right image corresponds to the X-ray spectra 35kVp, utilizing a Rh/Rh anode/filter combination, as illustrated in Figure 6A. The left image corresponds to X-ray spectra at 100 kVp, employing a W/Al anode/filter combination, as presented in Figure 6B.

**Figure 6:**
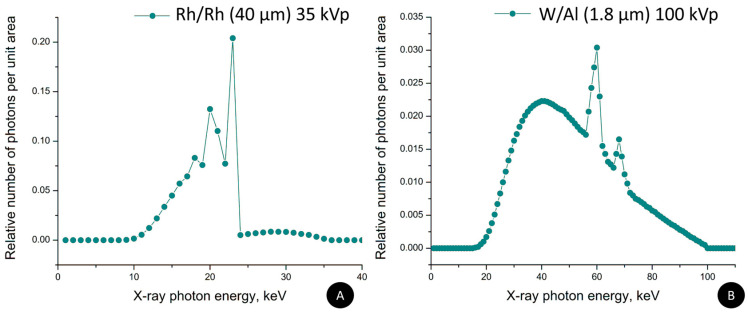
X-ray spectra used in the simulation: (**A**) Rh/Rh anode/filter combination at 35 kVp, (**B**) X-ray spectra of W/Al anode/filter combination at 100 kVp.

Figure 5 reveals the simulated X-ray images, acquired at both, lower and higher beam kVp (Figure 6). As expected, the increase of the kVp leads to diminished object contrast, which is due to the increased probability for Compton interaction at 100 kVp. In addition, the visual assessment of the images, depicted in Figure 5A,B, shows a very good resemblance in the object’s appearance for both, lower and higher kVp settings.

The elemental composition of both radiocontrast materials, Omnipaque and NaDITMB, were imported into the NIST XCOM web-based program [32], and obtained mass attenuation coefficients were approximated with the following equation:

(3) $$\frac{\mu \left(E\right)}{\rho }=e^{\left(-\lambda_{1}-\lambda_{2}.\log\;(E)\right)}+\lambda_{3}.{\left(\log\;(E)\right)}^{-1}+\lambda_{4}.{\left(\log\;\left(E\right).\log\;\left(E\right)\right)}^{-1}{\;+\;\lambda }_{5}$$

where *λ* parameters are detailed in Table 1 for both contrast compounds across two X-ray energy ranges, distinguished by the K-line of the iodine element.

The weighting coefficients w_NaDITMB_ and w_H2O_ in Equation (2) were defined as 4.47% and 94.93%, respectively. Correspondingly, for the Omnipaque solution, these coefficients were 5.03% and 94.97%, respectively.

**Figure 7:**
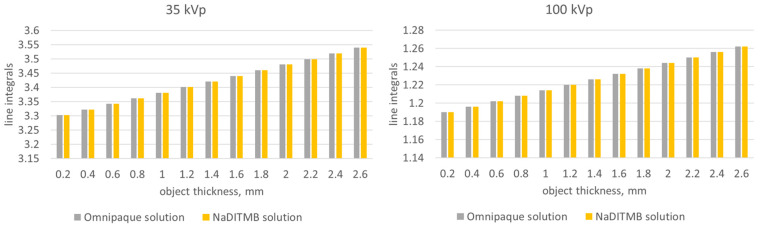
Values of the line integrals within the contrast objects of the phantom, shown in Figure 2A.

The subjective observations, based on Figure 5, are well supported by the results from the quantitative evaluation of the images, which included a comparison of measured line-integral values of the objects in the planar X-ray projections. Figure 7 summarizes these measurements for the studied contrast phantoms and the two incident X-ray beams. The findings from both lower (35 kVp) and higher (100 kVp) X-ray beams consistently demonstrate that the attenuation exhibited by the NaDITMB radiocontrast material precisely matches that of Omnipaque.

### 3.2. Experimental Study

**Figure 8:**
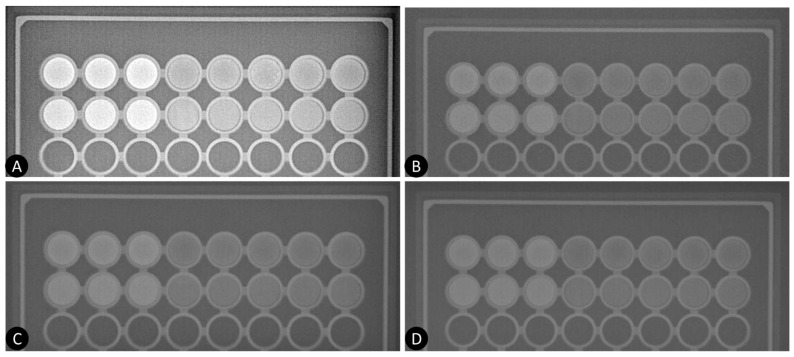
X-ray images of the contrast phantom at (**A**) 46 kVp, (**B**) 80 kVp, (**C**) 95 kVp, and (**D**) 100 kVp (from left to right). In each X-ray image, the first row represents the NaDITMB solution, while the second row depicts the Omnipaque solution.

Figure 8 shows the experimental radiographs of the plastic container with the radiocontrast agents at four different kVps: 46, 80, 95, and 100 kVp. As seen, increasing the kVp results in lower contrast of the images, which is due to the increase of the probability for Compton interactions. Further, the visual contrast of the new radiocontrast agent closely resembles that of the other contrast agent, as evidenced by the simulation study (Figure 5).

**Figure 9:**
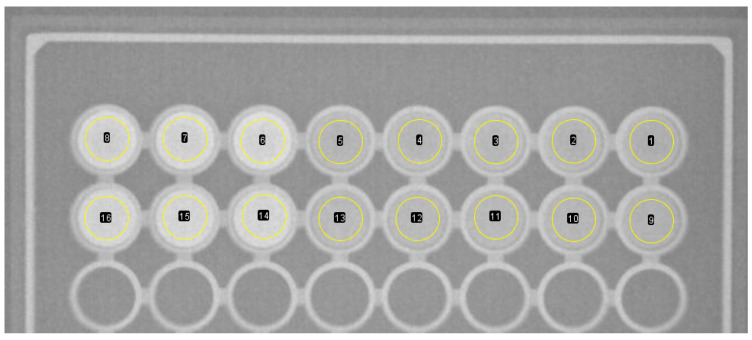
Grey value measurements of the imaged phantom. The selected area remains consistent for all iodine contrast agents and comprises 1124 pixels. Areas 1 to 5 represent 0.1 mL of NaDITMB, while areas 6 to 9 represent 0.2 mL of NaDITMB. Similarly, areas 9 to 13 correspond to 0.1 mL of Omnipaque, while areas 14 to 16 correspond to 0.2 mL of Omnipaque.

**Figure 10:**
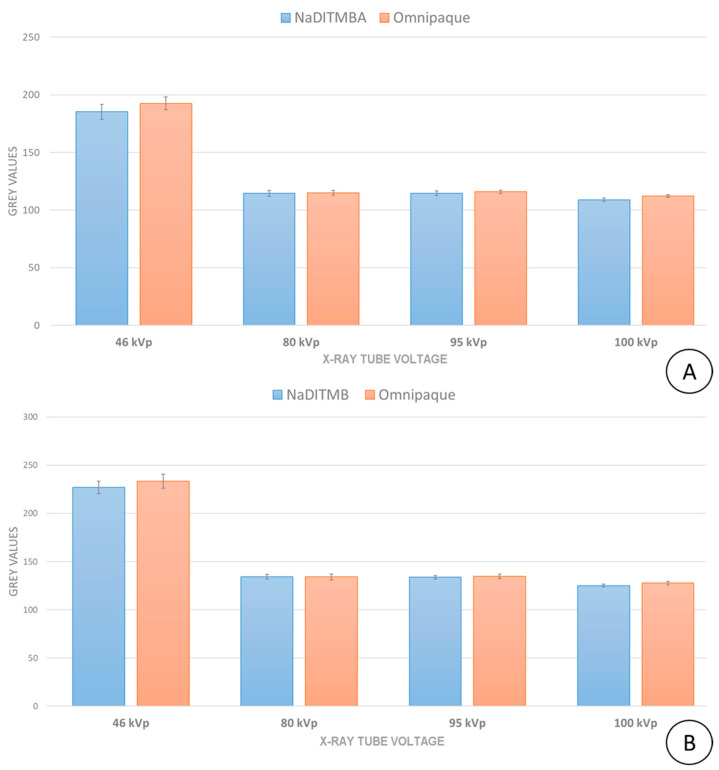
Measured grey values of the imaged radiocontrast materials: results for (**A**) 0.1 mL and (**B**) 0.2 mL contrast solution in the cylindrical cavities. The bars represent the mean values, while the error bars depict the standard deviations.

Figure 9 displays the implemented measurement of the grey values of the radiocontrast agents within the imaged plastic container. The selected area within the designated regions of interest for each contrast material corresponded to 1124 pixels, each measuring 148 μm × 148 μm. The summarized measurements are depicted in Figure 10.

The comparison of the grey values among the radiocontrast materials effectively validates the findings from the computational study. For all studied energies, the attenuation of X-rays by two contrast materials is very close. The maximum deviation between NaDITMB and Omnipaque corresponds to 3.7% for the X-ray tube voltage of 46 kVp and 0.1 mL quantity. For all other cases, this deviation was less than 2%, which is an excellent coincidence between simulated and experimental data.

In fact, 46 kVp represents the lower energy range of a general-purpose radiography machine, where the photon fluence may be inadequate to yield images with reduced fluctuations. These fluctuations are visible in the images as well, which is also responsible for the higher deviations from the mean value.

Future experimental investigation aims at the constructing of a calibration graph, reflecting the correlation between the concentration of the substances under investigation and their X-ray contrast behavior. It is anticipated that the conclusions drawn within this study will apply to higher iodine concentrations as well. Although simulations have validated this assumption, differences may arise in experimental outcomes, primarily attributed to the nature of the saturated solutions utilized.

The proposed radiocontrast agent offers an opportunity to be used as an alternative to the commercially available contrast media, especially for use in phantom studies dedicated to the optimization of novel breast imaging techniques, as well as demonstrating the superior capabilities of reconstruction algorithms and the potential for enhancements or the development of new acquisition geometries.

Real confirmation of the successful synthesis of NaDIТМB salt was obtained by means of ATR FTIR analysis. From the infrared spectrum presented in Figure A1, it can be seen that the absorption bands characteristic of the C=O group (at 1643 cm^−1^ and 1726 cm^−1^) completely disappear, and in its place, the appearance of two new bands is registered—these of asymmetric and symmetric COO^−^ stretching vibrations at 1583 cm^−1^ and at 1424 cm^−1^, respectively. Logically, the band characteristic of the v(O-H) vibrations is also absent in the spectrum of the studied salt. Furthermore, the unchanged number and position of all absorption bands inherent to aromatic C=C vibrations can be used as a further indicator of the conserved degree of substitution of the NaDITMB benzene “backbone”. Although of low intensity, oscillations for C-I bonds are also observed in the spectrum of the salt in question. On the other hand, the absence of iodide ions in the reaction volume (associated with the manifestation of an undesirable reaction of substitution of iodine atoms with hydroxyl groups) was further verified by the classical qualitative test with AgNO_3_.

The new radiocontrast agent NaDITMB was synthesized using a one-step experimental procedure. The latter can be considered relatively easy to perform, even by beginning chemists. The organic reactant used, TMBA, also known as eudesmic acid, is a natural compound, a constituent of *Eucalyptus* spp. [36]. Furthermore, the acid in question demonstrated the absence of cyto- and photoinitiated toxicity [25]. Despite all this, however, in order to qualify as a viable contrast agent for medical applications, the examined compound must successfully undergo comprehensive clinical safety assessments. Typically, this undertaking falls within the view of large pharmaceutical enterprises, given the substantial funding, extensive time, and specialized expertise required.

Moreover, DITMBA exhibits the unique ability to react spontaneously with bases and form water-soluble salts with them. On the other hand, the approximate price of the acid itself and its salts is comparable to that of other radiopaque substances—€5 per gram. Being regulated products, the Omnipaque^®^ cannot be freely purchased from pharmacies and utilized in experimental works. Converted into a form identical to that of the radiopaque medicines used, i.e., in a solution, the formed NaDITMB salt can be adequately radiographic assayed. This compound possesses the distinct advantage of being synthesizable in a single step, utilizing readily available starting materials. 

Another important aspect of this new contrast agent is its precisely identified composition and assured concentration, which makes it suitable for utilization with physical radiology phantoms; the latter is subjected to unrestrained exposures, thus achieving the goals of a given optimization task. Ensuring optimal performance is particularly crucial during the development of a prototype system, where the optimization task involves the type of detector used and the acquisition geometry in dual-energy contrast-enhanced mammography. This is essential to achieve the best level of performance for breasts with varying thicknesses and compositions [16,37]. Another important optimization task will concern the choice of reconstruction algorithm [38]. These phantoms offer a great possibility for X-ray technicians to undergo training, investigating the impact of kVp, exposure time, and anode current on the image quality of various anatomical structures.

## 4. Conclusions

This study highlights the promising potential of NaDITMB, a novel iodine-based material, as a radiocontrast agent for experimental studies employing physical phantoms. With a maximum deviation of 3.7% observed between NaDITMB and Omnipaque grey values at the lower energy of 46kVp—slightly below the typical range of 45-49 kVp utilized in contrast-enhanced breast applications—these findings are particularly relevant to medical imaging. Supported by comprehensive simulations, our experimental results provide robust validation. The results of this study will be used in the development and construction of physical phantoms designed for contrast-enhanced X-ray imaging.

## Figures and Tables

**Table 1:** The parameters λ of equation 3 required for calculating the mass absorption coefficients.

	*λ*_1_	*λ*_2_	*λ*_3_	*λ*_4_	*λ*_5_
[10:31.17) keV					
Omnipaque	33.7567	4.5735	−117.5345	−180.0997	0.9613
NaDITMB	32.8947	4.5145	−114.4335	−175.7637	1.0488
[31.17:150) keV					
Omnipaque	0.5985	1.9259	14.0620	11.3637	0.1039
NaDITMB	0.5286	1.9310	13.8811	11.1156	0.1016

**Figure A2:**
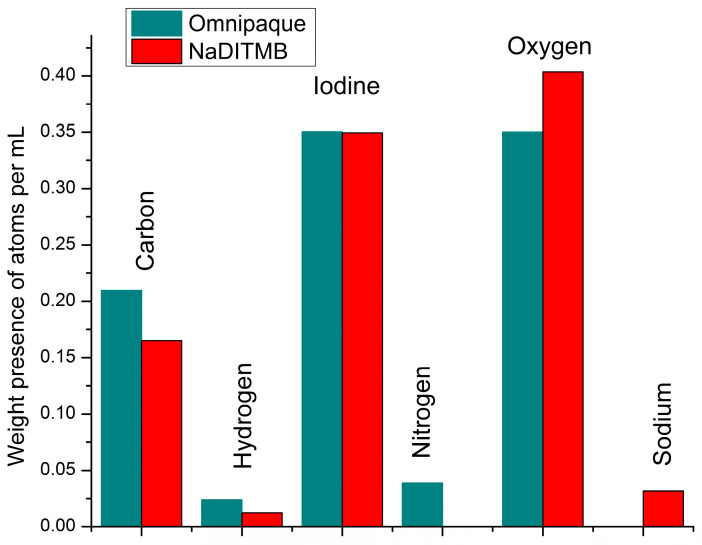
Elemental composition of the both working solutions—Omnipaque and NaDITMB.

## Data Availability

The data that support the findings of this study are openly available at http://doi.org/10.5281/zenodo.8296786, accessed on 12 December 2023.

## Appendix A

Figure A1 presents the ATR FTIR spectra of NaDITMB (black color) and DITMBA (red color), recorded through a Bruker Tensor II FTIR spectrometer in the 4000 ÷ 400 cm^−1^ spectral range. Figure A2 shows the elemental composition of the both working solutions—Omnipaque and NaDITMB.
